# Differences in ITO Surfaces According to the Formation of Aromatic Rings and Aliphatic Self-Assembled Monolayers for Organic Light-Emitting Diode Applications

**DOI:** 10.3390/nano11102520

**Published:** 2021-09-27

**Authors:** Myung-Gyun Baek, Sang-Geon Park

**Affiliations:** 1 Institute of Industrial Technology, Changwon National University, 20 Changwondaehak-ro, Uichang-gu, Changwon-si 51140, Gyeongsangnam-do, Korea; 20207232@changwon.ac.kr; 2 Department of Mechatronics Convergence, Changwon National University, 20 Changwondaehak-ro, Uichang-gu, Changwon-si 51140, Gyeongsangnam-do, Korea

**Keywords:** self-assembled monolayers, hole injection layer, organic light-emitting diodes

## Abstract

In this study, we investigated the effects on the characteristic changes in OLED devices of using self-assembled monolayers with different functional groups as the hole injection layer, resulting in changes in their performance. Thus, we confirmed that it is possible to control the wetting properties, surface roughness, and work function of the indium tin oxide (ITO) surface by introducing self-assembled monolayers (SAMs). The contact angle measurements confirmed that the substrate surface contact angle tended to increase with SAM deposition. In addition, AFM measurements confirmed that the substrate surface roughness tended to decrease when SAM was deposited on the surface. Finally, it was confirmed through the work function measurement results that the work function increased when the ITO surface was modified by SAM. Furthermore, compared to OLEDs using only the ITO anode, the SAM-modified device showed a higher current density (359.68 A/cm^2^), improved brightness (76.8 cd/cm^2^), and a smaller turn-on voltage (7 V). This approach provides a simple route for fabricating organic light-emitting diode applications.

## 1. Introduction

Organic light-emitting diodes (OLEDs) are self-illuminating displays that illuminate when an electrical current is applied. OLEDs have a fast response speed, wide viewing angles, full colors, high color reproducibility, and do not require a backlight, realizing thinner, lightweight, and flexible displays. These features also offer an advantage in terms of device price. However, obstacles remain, such as high production costs and product life expectancy, including burn-in based on material stability. Thus, global companies, research institutes, and universities continue to investigate solutions to these challenges [1,2,3,4].

An OLED is produced by placing a series of organic thin films between two electrodes. When a voltage is applied to the two electrodes, holes from the anode and electrons from the cathode are injected into the organic layers to be transported to the emissive layer. The transported electrons and holes recombine in the organic film and light that corresponds to the energy difference is then emitted. The multilayered organic thin films between the two electrodes in the OLED can resolve the differences in hole and electron mobility, and the ratio of the injected holes and electrons can be balanced to improve light emission efficiency. Because of its advantages, such as excellent hardness, chemical stability, high transparency, and low resistivity, indium tin oxide (ITO) is widely used as a material for anodes in existing OLED devices. However, ITO has low work function values compared to other organic materials used in manufacturing OLED devices [5,6]. The large differences in work function values between the organic film and ITO create a huge energy barrier that leads to a high turn-on voltage and low efficiency for OLED devices [7].

Many researchers have investigated methods to reduce the large injection barrier between the ITO and hole transfer material (HTM). Some of these include the use of Dipyrazino[2,3-f:20,30-h]quinoxaline-2,3,6,7,10,11-hexacarbonitrile (HAT–CN) [8], poly (3,4-ethylenedioxythiophene):poly(styrenesulfonate) (PEDOT:PSS) [9,10], and copper(ii) phthalocyanine (CuPc) [11]. One of these approaches involves the use of self-assembled monolayers (SAMs).

SAM refers to the organic monolayer that spontaneously forms on organic and inorganic surfaces and is organized into ordered domains. The molecular structure of a SAM includes the head group (which forms a chemical bond with the solid surface), alkyl chains in the middle, and functional groups that influence the function of the substrate surface.

No special equipment is required to deposit a SAM onto the substrate and the SAM is not influenced by the substrate shape or size. This enables the formation of an organic film on a substrate with a large area [12,13,14].

Typically, SAM has –SiCl_3_, –COOH, and –H_3_PO_4_ as the head groups, which chemically form on the substrate surface. In addition, the function of the deposited surface can vary depending on the type of functional groups that are introduced [15]. With these advantages, SAMs can be applied in many different fields [16,17,18,19,20]. Among them, alkylsilane-based SAMs are very useful for surface modification and functionalization applications because of their special stability [14].

SAM affects the function of the thin film depending on the functional group of the deposited material. Facilitating hole injection by introducing a SAM has been reported in various studies. For example, An et al. [21] reported that OLED performance improved when SAMs with different carboxylic acids were deposited onto ITO. Sharma et al. introduced a fluorine atom from a functional group to other sites to achieve an ITO work function in the range of 4.4–5.4 eV. Wang et al. [22] investigated the effect of the alkyl chain length of SAMs. Yang et al. [23] used 4-fluorothiophenol to form an Ag film on an ITO surface to improve hole injection and surface formation. Finally, M.G. et al. [24] conducted a study on the changes in ITO interface characteristics according to the formation of aromatic rings and aliphatic self-assembled monolayers.

The present study investigated the changes in OLED performance when using SAMs with different functional groups as the hole injection layer. Thus, in this study, trimethoxyphenylsilane (TTPS) and trimethoxy(naphthalene-2-yl)silane (TTNS) with the terminal functional group as the benzene ring, along with heptadecafluoro-1,1,2,2-tetrahydrodecyl)triethoxysilane (F10SAM) and 3,3,3trifluoropropyl) trimethoxysilane (F3SAM) with a trifluoromethyl group were used as sample hole injection layers to determine their effects on the properties of OLED devices. 

## 2. Experimental

### 2.1. Materials

ITO, used as the positive electrode, was purchased from AMG Co., Ltd. (Prides Crossing, MA, USA). The as-purchased ITO was deposited with a thickness of 150 ± 10 nm, with a sheet resistance value of ≤10 Ω/sq, a transmittance of ≥85% at *λ* = 550 nm. SAMs deposited on the surfaces of ITOs included TTPS, TTNS, F-3SAM, and F10SAM and were supplied by Gelest (Bucks County, PA, USA). *N*,*N*.-diphenyl-*N*,*N*.-bis(1-naphthyl)-1,1′-biphenyl-4,4′-diamine (NPB), 2,3,6,7-Tetrahydro-1,1,7,7,-tetramethyl-1H,5H,11H-10-(2-benzothiazolyl)quinolizino[9,9-a,1gh]coumarin (C545T), and tris (8-hydroxyquinoline)aluminum (Alq_3_) were supplied by TCI Chemicals (India) Pvt. Ltd. (Chennai, India) Finally, LiF from Advance Chemicals, Ltd. (Port Coquitlam, BC, Canada) and 99.999% pure Al from Alfa Aesar were also used.

### 2.2. Substrate Preparation

The debris from the surface of the ITO substrate was removed via ultrasonic cleaning; i.e., using deionized water, followed by acetone, deionized water, and 2-propanol for 5 min. Annealing was then performed for 5 min with a hot plate at 150 °C to remove residual moisture from the surface. The final cleaning was performed on the dried ITO substrate via UV ozone treatment for 20 min to remove organic matter from the surface.

### 2.3. SAM Modification of the ITO Surface

**Figure 1:**
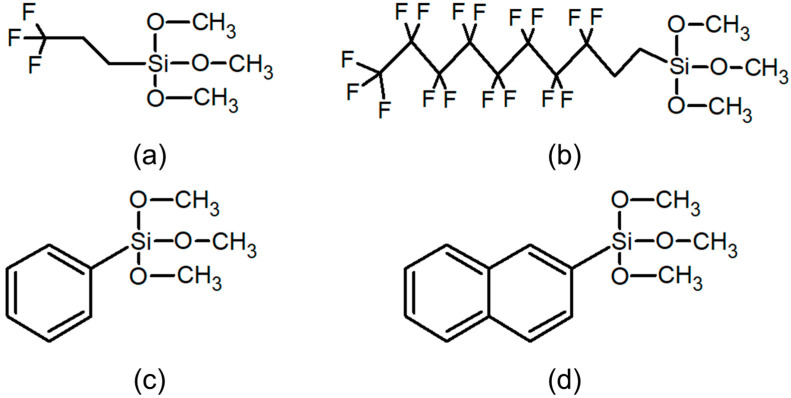
Molecular structures of SAMs: (**a**) (F3SAM), (**b**) F10SAM, (**c**) TTPS, (**d**) TTNS.

To evaluate OLED performance when SAMs with different functional groups were used as the hole injection layer, TTPS and TTNS with a benzene ring, and F3SAM and F10SAM with chain structures were used in this study. The molecular structures of these chemicals are shown in Figure 1. The deposition of SAMs on the ITO substrate was performed using a dipping process. The solution for the dipping process was prepared by adding SAMs to 100 mL of ethanol until the concentration reached 2 mM. The solution was stirred at 300 rpm for 20 min. The cleaned ITO substrate was then immersed in the solution at room temperature for 72 h to perform SAM deposition. The deposited ITO was ultrasonically cleaned for 5 min and finally annealed on a hot plate at 150 °C to complete the SAM deposition process. 

### 2.4. Device Fabrication

The assembly of the full device was completed using a SAM-modified ITO in a vacuum thermal evaporator placed in a nitrogen glove box. At 699 μPa, 100 nm of the NPB layer was deposited, followed by 40 nm of Alq_3_:C545T, 25 nm of Alq_3_, 1 nm of LiF, and, finally, 120 nm of aluminum cathode.

### 2.5. Monolayer and Device Characterization

The contact angle was measured using the sessile drop method with UNI-CAM from GIT Tech, Ltd. (Ansan, Korea) Atomic force microscopy (AFM) was performed with an XE-100 (Park Systems, Ltd., Suwon, Korea) in non-contact mode. NCHR (Park Systems Co., Ltd.) was used as the tip (resonance frequency 330 kHz, tip length, 10~15 µm, tip radius typical, <10 nm max, tip coating Backside reflex coating (Al)), and ultraviolet photoelectron spectroscopy (UPS) was performed using HIS 13 from FOCUS GmbH, Ltd. (Huenstetten, Germany), with He as the discharge source. Finally, the electrical optical properties of the OLED were measured using a spectral camera PR650 from Photo Research, Ltd. (Los Angeles, CL, USA). 

## 3. Results

**Figure 2:**
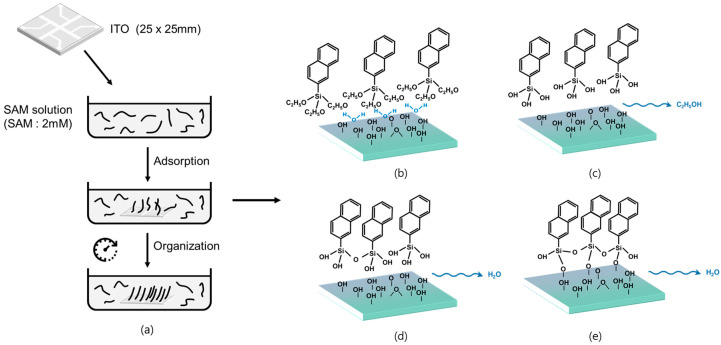
SAM formation process and principles: (**a**) SAM formation principle, (**b**) physisorption, (**c**) hydrolysis, (**d**) covalent grafting to the substrate, (**e**) in-plane reticulation.

This section describes the mechanism of SAM formation on the surface of an ITO substrate immersed in a solution. The following stages occurred during the formation process: (1) a low-density stage, in which a low SAM coverage was present on the substrate and exhibited fluidity; (2) an intermediate stage, in which the SAM coverage was greater than the threshold and the SAM molecules aligned horizontally on the surface to cause uneven condensation; and (3) a final high-density stage, in which the SAM was rearranged perpendicular to the substrate, resulting in a well-aligned SAM [13,14,24]. The hydroxyl group formed on the surface of the hydrated ITO substrate to produce a hydrophilic surface and, as the SAM deposition proceeded, the polar head group of the SAM became increasingly hydrolyzed to form hydroxyl groups. The hydrolyzed SAM dissolved into the water layer on the ITO substrate and the head groups formed hydrogen bonds with hydroxyl groups near the surface. Finally, a condensation reaction occurred, forming H_2_O from the hydrogen bond and linking the head groups with those on the surface. Accordingly, cross-packing occurred, while a strong chemical bond with the surface was achieved for successful deposition on the surface [25,26]. The head group of the SAM reacted with the –OH group from the ITO substrate to form a Si–O–Si covalent bond and the properties of the ITO surface changed depending on the functional groups of the SAM. This process is illustrated in Figure 2.

**Figure 3:**
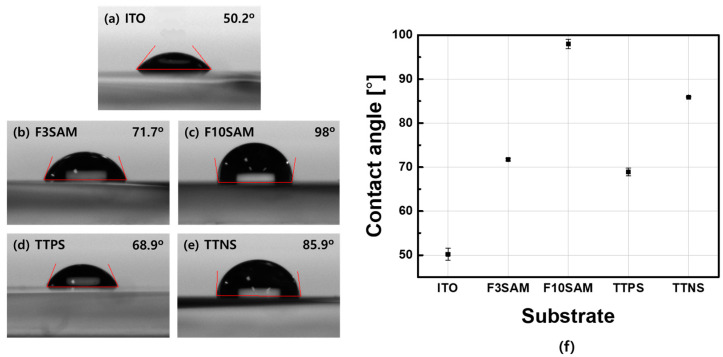
Contact angle measurements of SAMs on ITO: (**a**) ITO, (**b**) TTPS, (**c**) TTNS, (**d**) F3SAM, (**e**) F10SAM, (**f**) contact angle graph.

To evaluate whether each SAM was successfully adsorbed onto the ITO substrate, the contact angles for each SAM were measured. The results are shown in Figure 3. The contact angles for ITO, TTPS, TTNS, F3SAM, and F10SAM were 50.2°, 68.9°, 85.9°, 71.7°, and 98°, respectively. The results indicate that the SAMs were successfully adsorbed onto the ITO surface and confirmed that SAM deposition onto the ITO surface produced a relatively high contact angle because of the characteristics of the functional group. For the benzene ring functional group, the contact angle increased as the number of benzene rings increased and, for the aliphatic groups, the contact angle increased with increasing alkyl chain length. In addition, the modified ITO surface via SAM with a chain structure was shown to have a larger contact angle than the SAM with a benzene ring.

**Figure 4:**
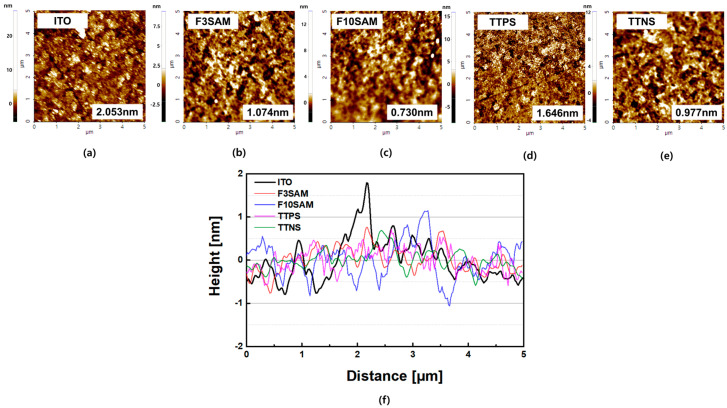
AFM measurements of SAMs on ITO: (**a**) ITO, (**b**) F3SAM, (**c**) F10SAM, (**d**) TTPS, (**e**) TTNS, (**f**) SAM roughness.

Figure 4 shows the AFM image scanned at 5 μm × 5 μm area and Table 1 lists the Root–mean–square (RMS) surface roughness results for the ITO surface with different deposited SAMs. AFM revealed that the RMS surface roughness values of ITO were 2.053 nm, while those of TTPS, TTNS, and F3SAM were 1.646 nm, 0.997 nm, and 1.074 nm, respectively. The AFM measurement results indicated that SAMs deposited onto the ITO surface reduced the RMS surface roughness and revealed surface topologies, with one or more SAMs exhibiting single monomolecular layers formed on the ITO surface [27,28]. When fabricating OLED devices, a large surface roughness of the ITO surface leads to a short lifetime of OLED devices and an increase in the turn-on voltage due to the poor step coverage of organic matter deposited on ITO surfaces; i.e., the uniform roughness of ITO surfaces achieved by SAM deposition can remove the step coverage between the ITO surface and the hole transport layer, thereby improving the efficiency of OLED devices.

**Figure 5:**
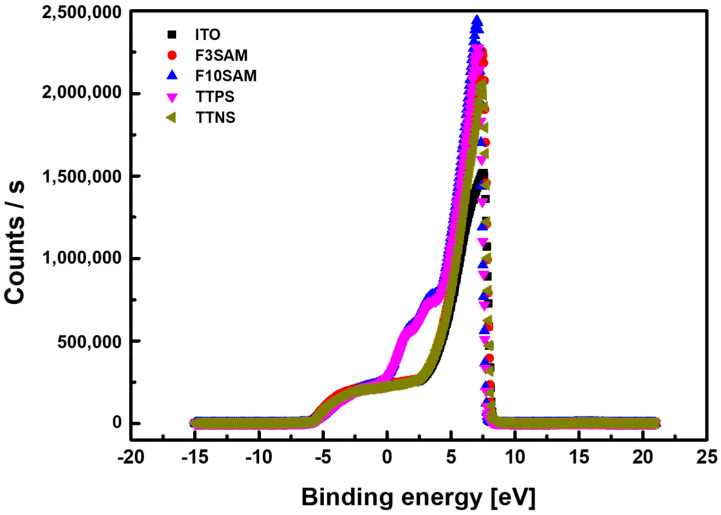
Results of work function UPS measurements.

Figure 5 shows the work function graph. Table 2 presents the measurement results for the ITO surface with different deposited SAMs. The work function was obtained through UPS and can be expressed as

*ϕ* = *hv* − (*E_cutoff_* − *E_F_*) (1)

where *ϕ* represents the work function value, *hv* is the light energy from the He incident light, *E_cutoff_* is the secondary electron cutoff energy, and *E_F_* is the Fermi level for the investigated sample [29]. The UPS measurement results revealed an increase in the work function on the ITO surface with SAM deposition. This change could lead to increased efficiency through a greater hole injection by bridging the work functions between ITO and HTM. 

**Figure 6:**
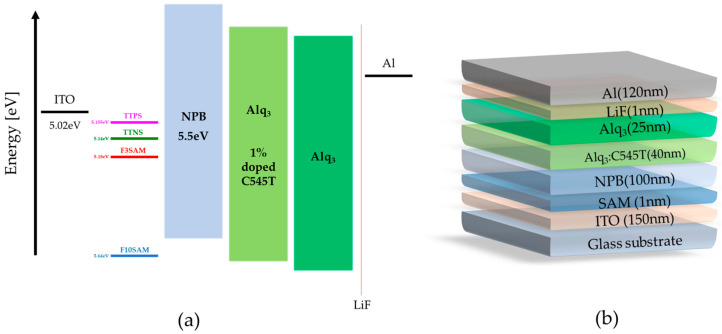
(**a**) Energy levels of the device, (**b**) OLED device structure.

To verify the electrical and optical properties of the OLED devices when SAMs were deposited on the ITO surface, the entire OLED device was fabricated using the vacuum heat deposition method. The fabricated OLED structure is illustrated in Figure 6.

The structure of the device included ITO/NPB (100 nm)/Alq_3_:C545T (40 nm)/Alq_3_ (25 nm)/LiF (1 nm)/Al (120 nm). OLED devices were fabricated with varying SAMs (placed between the ITO and NPB), and the characteristics of each device were analyzed. 

**Figure 7:**
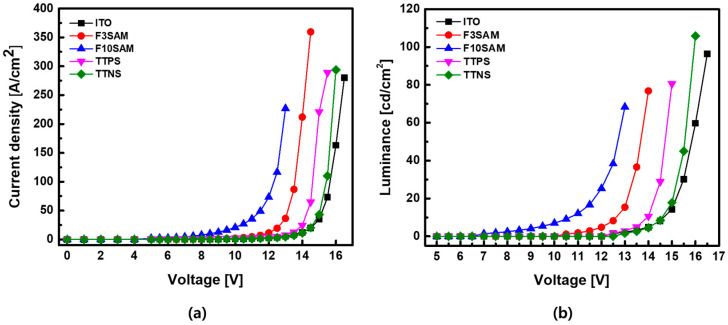
OLED characteristics: (**a**) J–V curve, (**b**) L–V curve.

Figure 7 presents the electrical and optical characteristics of the OLED devices when different SAMs are used as the hole injection layer. Figure 7a,b show the current density and luminance, respectively, both as functions of voltage. For the basic device that was ITO-only and for the device with SAM, the turn-on voltages were approximately 12.5 V and 10–12 V, respectively. For F10SAM, the turn-on voltage was reduced to approximately 7 V. These results suggest that the addition of a SAM between the ITO and HTM could reduce the turn-on voltage of OLED devices. For the basic ITO-only device, the current density and luminance were 280.1 A/cm^2^ and 96.3 cd/m^2^, respectively, while the current density and luminance of F3SAM were 359.7 mA/cm^2^ and 76.8 cd/m^2^, respectively.

These results indicate that introducing a SAM as the hole injection layer can lead to a decrease in the turn-on voltage and can increase the current density and luminance of OLED devices.

## 4. Conclusions

This study investigated the effects of using SAMs with different functional groups as the hole injection layer on the characteristic changes in OLED devices that can lead to changes in their performance. The contact angle measurement results for ITO, F3SAM, F10SAM, TTPS, and TTNS were 50.2°, 71.7°, 98°, 68.9°, and 85.9°, respectively, and an increase in the surface contact angle was observed with SAM deposition. In addition, the RMS surface roughness results were obtained through AFM and were as follows: F3SAM (1.074 nm), F10SAM (0.730 nm), TTPS (1.646 nm), and TTNS (0.977 nm). When a SAM was deposited on the surface, a decrease in the RMS surface roughness was observed. Finally, the work function values for ITO, F3SAM, F10SAM, TTPS, and TTNS were 5.08 eV, 5.18 eV, 5.64 eV, 5.105 eV, and 5.14 eV, respectively, indicating that an increase in the work function occurred when the ITO surface was modified by the SAMs. These results suggest that for a SAM with an aliphatic structure, the van der Waals interaction between alkyl chains led to an aligned organic thin film. As a result, better aligned organic films were obtained with F10SAM than with F3SAM, leading to high contact angles, low RMS surface roughness values, and high work function values. However, with aromatic ring structures, the number of π electrons increased with an increase in the number of aromatic rings, leading to a decrease in the energy level. Finally, a decrease in the turn-on voltage and an increase in the current density and luminance were observed for OLED devices using a SAM as the hole injection layer. 

These results seem to be derived from the fact that when SAM was used as the hole injection layer, it acted as a ladder for the carriers to overcome the hole injection barrier between ITO and HTM, facilitating the hole injection process to a considerable extent.

## Figures and Tables

**Table 1:** Results of AFM measurements.

Material	Min (nm)	Max (nm)	Mid (nm)	Rpv (nm)	Rq (nm)	Ra (nm)
ITO	−5.407	27.411	11.002	32.818	2.053	1.342
F3SAM	−4.735	9.409	2.337	14.144	1.074	0.842
F10SAM	−2.943	14.007	5.532	16.951	0.73	0.535
TTPS	−8.624	15.994	3.685	24.618	1.646	1.262
TTNS	−4.346	12.163	3.908	16.509	0.977	0.753

(Rpv: R peak-to-peak value; Rq: R root mean square; Ra: R average).

**Table 2:** Results of UPS measurements.

Material	*hv*	*E_cutoff_*	*E_F_*	*ϕ*
ITO	21.21 eV	2.303 eV	9	5.08 eV
F3SAM	21.21 eV	8.21 eV	9	5.18 eV
F10SAM	21.21 eV	7.75 eV	9	5.64 eV
TTPS	21.21 eV	8.285 eV	9	5.105 eV
TTNS	21.21 eV	8.25 eV	9	5.14 eV

(*hv*: light energy; *E_cutoff_*: secondary electron cutoff energy; *E_F_*: Fermi level; *ϕ*: work function).
